# Conformational Properties and Putative Bioactive Targets for Novel Thiosemicarbazone Derivatives

**DOI:** 10.3390/molecules27144548

**Published:** 2022-07-16

**Authors:** Nikitas Georgiou, Antigoni Cheilari, Danai Karta, Eleni Chontzopoulou, Janez Plavec, Demeter Tzeli, Stamatia Vassiliou, Thomas Mavromoustakos

**Affiliations:** 1 Laboratory of Organic Chemistry, Department of Chemistry, National and Kapodistrian University of Athens, Panepistimioupolis Zografou, 11571 Athens, Greece; nikitage@chem.uoa.gr (N.G.); danaikarta@gmail.com (D.K.); elenichontzo@chem.uoa.gr (E.C.); 2 Department of Pharmacognosy and Natural Products Chemistry, Faculty of Pharmacy, National and Kapodistrian University of Athens, Panepistimiopolis Zografou, 15771 Athens, Greece; cheilarianti@pharm.uoa.gr; 3 Slovenian NMR Centre, National Institute of Chemistry, SI-1001 Ljubljana, Slovenia; janez.plavec@ki.si; 4 Laboratory of Physical Chemistry, Department of Chemistry, National and Kapodistrian University of Athens, Panepistimioupolis Zografou, 11571 Athens, Greece; tzeli@chem.uoa.gr; 5 Theoretical and Physical Chemistry Institute, National Hellenic Research Foundation, 48 Vassileos Constantinou Ave., 11635 Athens, Greece

**Keywords:** thiosemicarbazones, NMR spectroscopy, quantum mechanics, molecular binding, DFT

## Abstract

The structure assignment and conformational analysis of the thiosemicarbazones, **DKI21** and **DKI24**, were performed through homonuclear and heteronuclear 2D Nuclear Magnetic Resonance (NMR) spectroscopy (2D-COSY, 2D-NOESY, 2D-ROESY, 2D-HSQC, and 2D-HMBC) and quantum mechanics (QM) calculations, using Functional Density Theory (DFT). In addition, utilizing a combination of 2D-NOESY and 2D-ROESY spectra an *exo* structure was established for both of the analogs. This experimental results were confirmed by theoretical mechanistic studies, as the lowest minima conformations derived through DFT calculations were compatible with the spatial correlations observed in the 2D-NOESY and 2D-ROESY spectra. Finally, molecular binding experiments were performed to detect the potential targets for **DKI21** and **DKI24**, derived from SwissAdme. *In silico* molecular binding experiments showed favorable binding energy values for the most of the enzymes studied. The ADMET calculations, using the preADMET and pKCSm software, showed that the two molecules appear as possible drug leads.

## 1. Introduction

Thiosemicarbazones represent a class of small molecules with various pharmacological properties [1], including antiviral [2], antibacterial [3], and antitumor activities [4]. Moreover, they represent key intermediates for a great variety of heterocyclic products, such as thiazolidine-4-ones.

Recently, the interest in thiazolidine-4-one derivatives has been increased among scientists due to their broad spectrum of biological activities, including antidiabetic, antibacterial, antifungal, anticancer, and anti-inflammatory, confirmed by numerous reviews on the activity and mechanisms of action of thiazolidine-4-ones [5,6,7,8,9].

The main synthetic route to thiazolidine-4-ones is the reaction between thiosemicarbazones and α-halo carboxylic esters, with several interesting structures obtained recently with this method [10,11,12,13].

However, the regio-outcome of this reaction has never been studied in detail, to the best of our knowledge, with the exception of Qian [14] who, apart from the expected thiazolidine-4-one as a major product, observed its regio-isomeric thiazolone and isolated the two regio-isomers as N-benzyl derivatives. This study triggered our research interest to perform a detailed study towards the refinement of this reaction regio-selectivity. In addition, a main target of our research was to unambiguously assign the regio-isomeric product through 2D-NOESY and 2D-ROESY spectroscopy methodologies and explain the product by theoretical means.

**Scheme 1:**
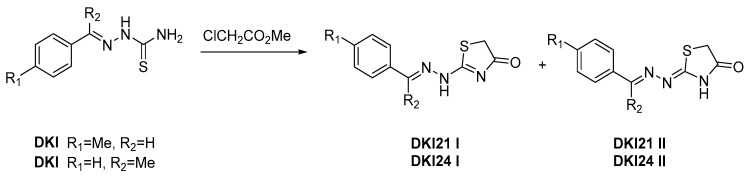
These molecules underwent structure elucidation, utilizing 2D-NOESY and ROESY experiments, to establish their *exo* or *endo* products. In addition, molecular docking experiments were performed to establish the putative bioactive targets.

Thiosemicarbazones DKI reacted with methyl chloroacetate in ethanol in the presence of fused sodium acetate to give thiazolone/thiazolidinone derivatives, **DKI21** and **DKI24,** as a mixture of regio-isomers **I** and **II** (Scheme 1). The geometric configuration of the double bond and the regio-chemistry of the thiazolylhydrazones are the topic of this research work.

## 2. Results

### 2.1. Structure Assignment

**Figure 1:**
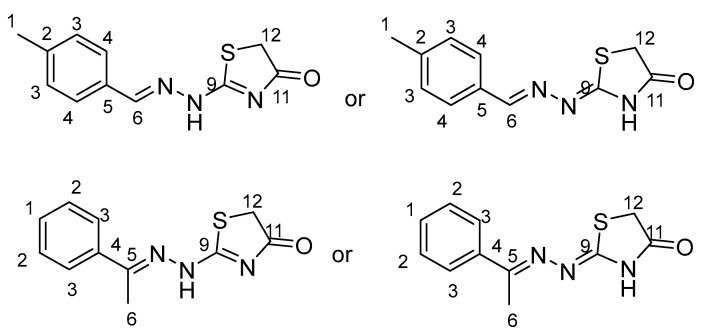
Possible Structures of **DKI21** and **DKI24**.

The possible structures of **DKI21** and **DKI24** are shown below in Figure 1.

The methyl group H-1 is a convenient starting point for the structure assignment of **DKI21**, which resonates at 2.35 ppm. Through 2D-NOESY and 2D-COSY, H-3 and H-4 are identified. Through 2D HSQC, the H-3 and H-4 show ^1^J_C-H_ coupling with the C3 and C4, correspondingly, and, therefore, C3 and C4 are assigned unambiguously at 129.9 and 128.10 ppm, respectively. Through 2D-NOESY, H-6 is identified, because it shows spatial correlation with the H-4. H-12 is identified, as the remaining unidentified proton. Through 2D-HSQC, all of the carbons were identified, except for the quaternary and carbonyl ones. These carbons were identified through 2D-HMBC. Specifically, H-1 shows ^2^J_C-H_ with C-2, H-4 shows ^2^J_C-H_ with C-5, H-12 shows ^2^J_C-H_ with C-11, and finally, H-12 shows ^3^J_C-H_ with C-9. Based on this strategy, the complete identification of all of the proton and carbon atoms of the **DKI21** molecule was achieved. The same procedure was achieved for the isomer **DKI24** molecule. The two identification strategies, with all of the spectra obtained, are shown in detail in the Supplementary Materials.

Table 1 shows the proton chemical shifts of these compounds, their bond and spatial correlations, and heteronuclear correlations.

### 2.2. Conformational Analysis

**Figure 2:**
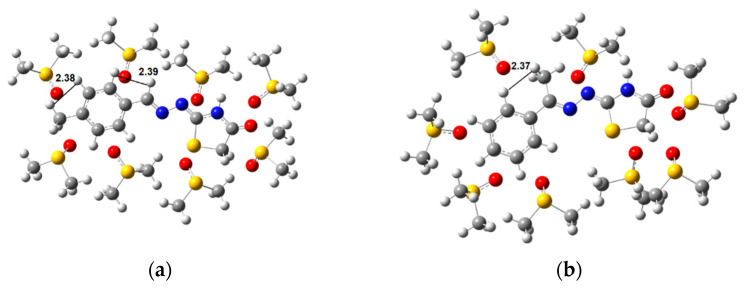
The lowest in energy conformations for **DKI21** (**a**) and **DKI24** (**b**) and their critical spatial correlations that determine their lowest energy conformations.

DFT was used to predict the lowest energy conformations for **DKI21** and **DKI24**. Various structures were used as the initial guess for optimization. The lowest conformations for **DKI21** and **DKI24** are shown below. Considering the predicted energy values, the structures of the compounds, and the correlations that were observed in the 2D-NOESY and 2D-ROESY spectra, the structures shown in (a) and (b) are taken as the most probable conformations for **DKI21** and **DKI24**, respectively (Figure 2).

**Figure 3:**
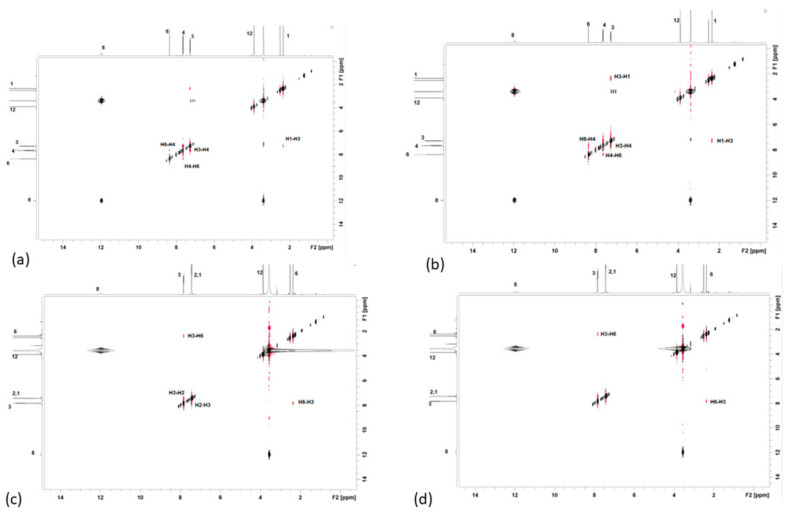
2D–NOESY spectra for **DKI21** (above) using d8 = 0.15 s (**a**) and 0.30 s (**b**) and **DKI24** using d8 = 0.15 s (**c**) and d8 = 0.30 s (**d**) (bottom). The spectra were recorded in DMSO-d_6_ on a Bruker AC 500 MHz spectrometer at 25 °C.

The correlations between the protons in 2D-NOESY, using mixing times 0.3 and 0.15 s, are shown below. These correlations were confirmed, using other mixing times and 2D-ROESY experiments (Figure 3).

According to the results, the *exo* compound is the one that matches with the experimental results. The reason for this is that it does not observe the correlation between the amino group (NH) and the H-6 in each compound.

Furthermore, the *exo* intermediate has a low energy barrier, due to the extended resonance forms that *exo* can undergo. These extending resonance forms cannot be applied for the higher energy *endo* analogue. In addition, in the **DKI24** *exo* intermediate there is a hydrogen bond between proton 6 and nitrogen, while it does not favor in *endo*. Finally, in the **DKI24** exo conformation the atoms 3, 4, 5, and 6 are at the same level, in contrast with the *endo* conformation. On the other hand, in **DKI21**, the *exo* intermediate is the most favorable because there is a repulsion between the amino proton and proton in carbon 6, that does not exist in *exo*.

Regarding the molecular orbitals of both the *endo* and *exo* conformations of both of the compounds, to derive a more quantitative answer, the HOMO molecular orbital is localized in the heterocyclic ring, while the LUMO molecular orbital is mainly localized in the aromatic ring and in the nitrogen atoms. The frontiers’ molecular orbitals are depicted in Figures S19 and S20 (Supplementary Materials).

Finally, the hardness (η), chemical potential (μ), and softness (S) of the lowest in the energy conformers were calculated from the energies of frontier HOMOs and LUMOs [15,16]. The following equations are used: η = ((LUMO) − ε(HOMO))/2;μ = (ε(LUMO) + ε(HOMO))/2; S = 1/η. It is found that the *exo* conformers present a lower H–L energy gap than the *endo* ones, and, thus, lower hardness and higher chemical softness, see Table S1 (Supplementary Materials). That shows that the *exo* intermediates are more reactive than *endo* intermediates. The energetics are given in the Supplementary Materials.

### 2.3. Reaction Mechanism

Using the DFT calculations, we have studied the reaction mechanism to confirm the experimental results. The first intermediate to study was the formation of the *endo* and *exo* double bond in the **DKI21** compound.

**Figure 4:**
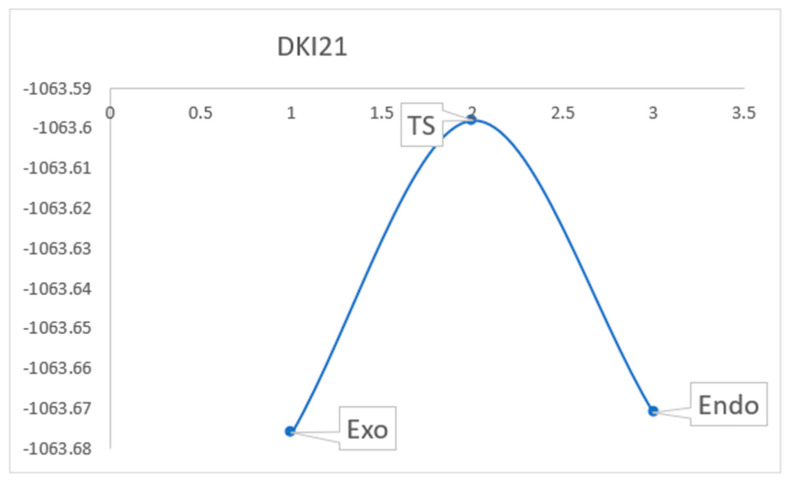

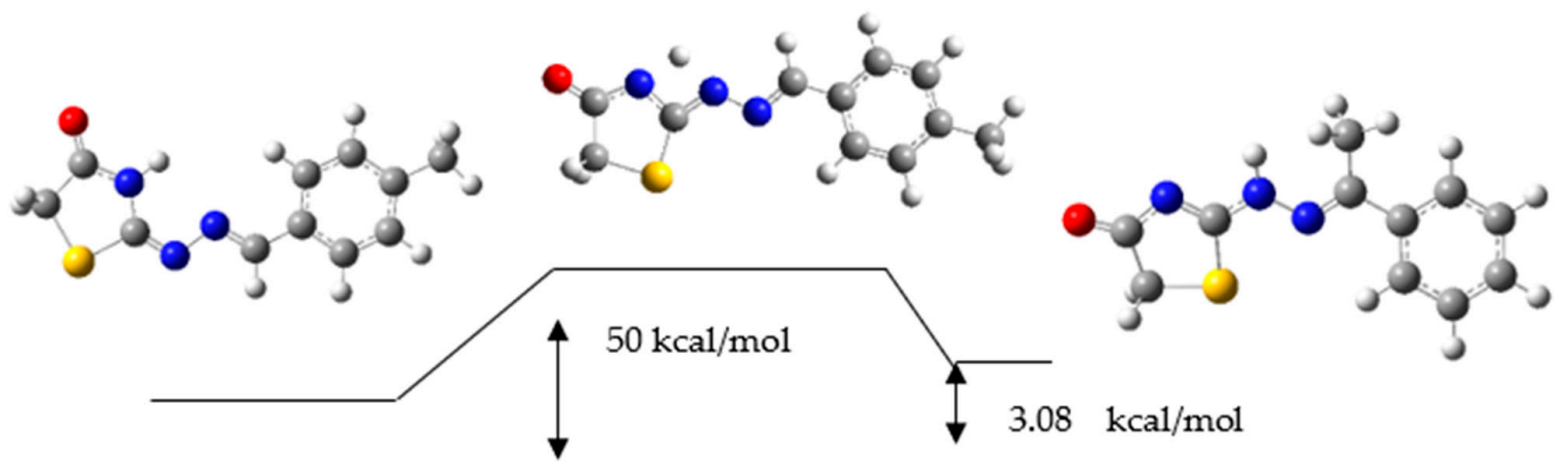
Energy diagram (in Hartree) of the *endo* and *exo* isomerization of the **DKI21** compound.

An energy diagram of the formation between the *endo* and *exo* is shown below (Figure 4).

**Figure 5:**
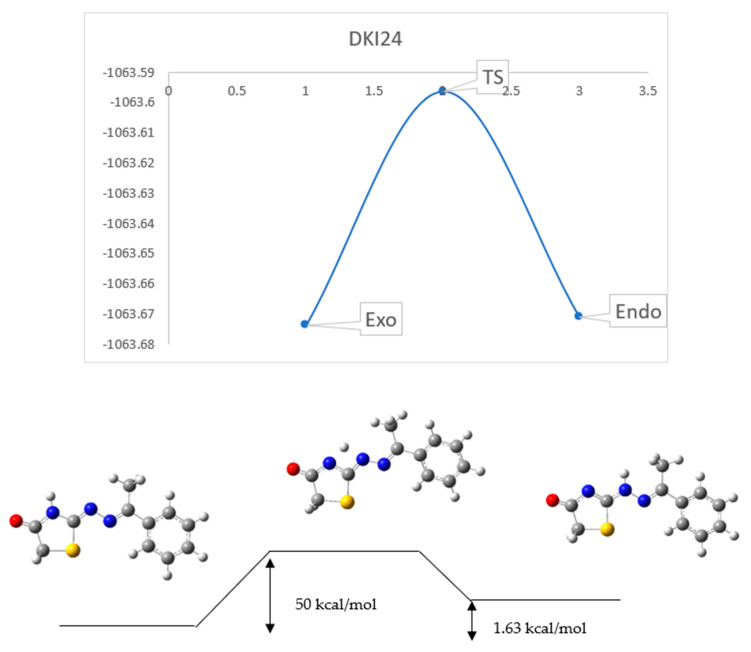
Energy diagram (in Hartree) of the *endo* and *exo* isomerization of the **DKI24** compound.

The next intermediate to study was the formation of the *endo* and *exo* double bond in the **DKI24** compound (Figure 5).

An energy diagram of the formation between *endo* and *exo* is shown below.

It becomes clear from the above Figures that the *exo* intermediate compound has a very low energy barrier and it is therefore a preferable pathway. This comes in agreement with the NMR experiments.

### 2.4. Molecular Binding

SwissTarget Prediction was used to predict some of the possible macromolecules for the *in silico* experiments. Eight targets were detected, specifically, the monoamine oxidase B (MAO B), the Melatonin Receptor MT1, the Human acid Ceramidase, the Kinase Domain, the ADAMTS5, the Cell division cycle 7-related protein kinase/Activator of S phase kinase, the Phosphodiesterase 4D, and the Lipoxygenase-1. The grid parameters used were the same for all of the substrates.: X = 40; Y = 40; Z = 40 (default); and the distance of the dots: 0.375 Å (default).

Then, the coordinates from the co-crystallized ligand, which were used for the active center of each macromolecule, were: 2VRM [17]: X = 53.541; Y = 151.302; Z = 23.411 6ME2 [18]: X = 24.378; Y = −21.359; Z= 46.386, 6MHM [19]: X = −7.583; Y = 4.843, Z = 28.586; 5HIE [20] X = 71.977; Y = 12.669, Z = 131.026, 3G4L [21]: X = 20.794; Y = −4.795; Z = 28.848; 4F9C [22]: X = 17.309; Y = 21.398; Z = 58.968 and 2RJQ [23]: X = −42.728; Y = −22.678; Z = 6.105.

The results are shown in Table 2.

**Figure 6:**
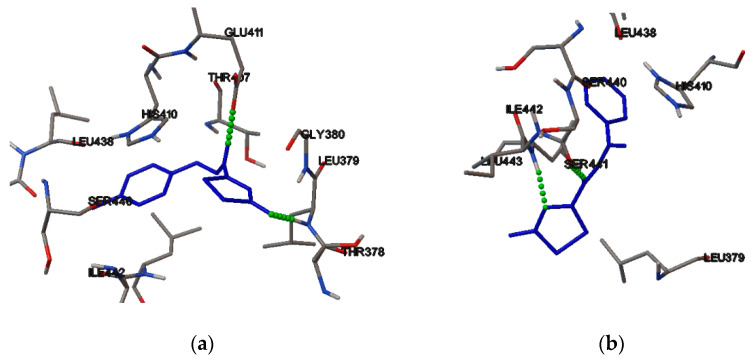
Interactions of **DKI21** (**a**) and **DKI24** (**b**) with ADAMTS5, which gave the most favorable results.

The most favorable target was found to be the ADAMTS5 enzyme. The binding energy and the inhibition constants were calculated computationally via the AutoDock program. Thus, these compounds can serve as potential leads for these targets. The interactions of these two molecules with ADAMTS5 are shown in Figure 6. Both of the compounds form two hydrogen bonds. Specifically, **DKI21** forms one hydrogen bond with GLU411 amino acid and one hydrogen bond with THR378. In addition, **DKI24** forms one hydrogen bond with SER441 amino acid and one hydrogen bond with ILE442. Both of the compounds bind favorably to the active center of the enzyme.

**Figure 7:**
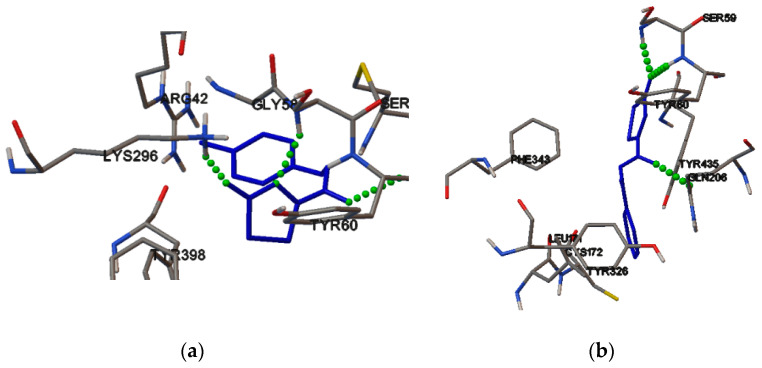
Interactions of **DKI21** (**a**) and **DKI24** (**b**) with Monoamine oxidase B.

The interactions of these two molecules with monoamine oxidase B are shown in Figure 7. Specifically, **DKI21** forms three hydrogen bonds with the TYR60, GLY68, and ARG42 amino acids. In addition, the **DKI24** forms two hydrogen bonds with SER206 and SER50 amino acids. Both of the compounds bind to the active center of the enzyme and the **DKI24** binds more favorably than the **DKI21**.

**Figure 8:**
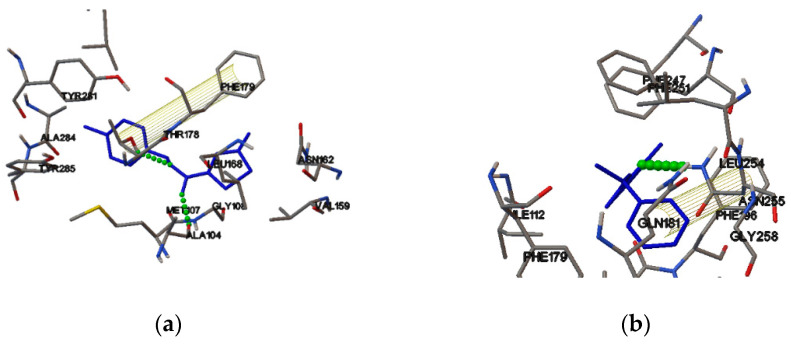
Interactions of **DKI21** (**a**) and **DKI24** (**b**) with Melatonin receptor MT1.

The interactions of these two molecules with the Melatonin receptor, MT1, are shown in Figure 8. Specifically, the **DKI21** forms two hydrogen bonds with the TYR175 and ALA104 amino acids and one pi–pi interaction with the PHE179 amino acid. In addition, the **DKI24** forms one hydrogen bond with LEU254 amino acid and one pi–pi interaction with the PHE196 amino acid. Both compounds bind favorably to the active center of the enzyme.

**Figure 9:**
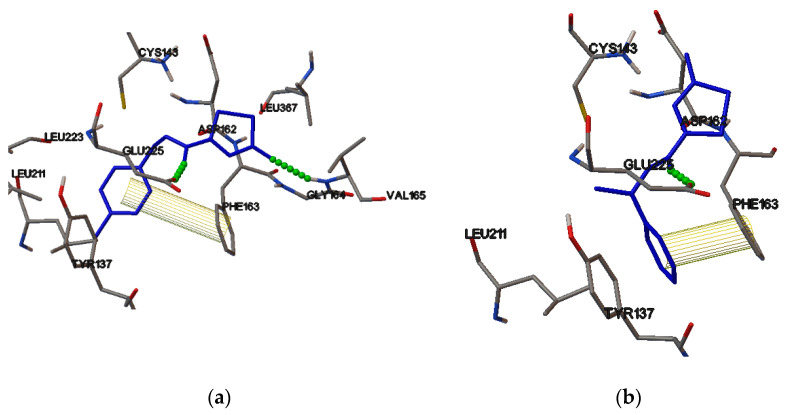
Interactions of **DKI21** (**a**) and **DKI24** (**b**) with Human acid ceramidase.

The interactions of these two molecules with the Human acid ceramidase are shown in Figure 9. Specifically, the **DKI21** forms two hydrogen bonds with GLU225 amino acid and with GLY164, and one pi–pi interaction with PHE163 amino acid. In addition, the **DKI24** forms one hydrogen bond with the GLU225 amino acid and one pi–pi interaction with PHE163 amino acid. Both of the compounds bind favorably to the active center of the enzyme.

**Figure 10:**
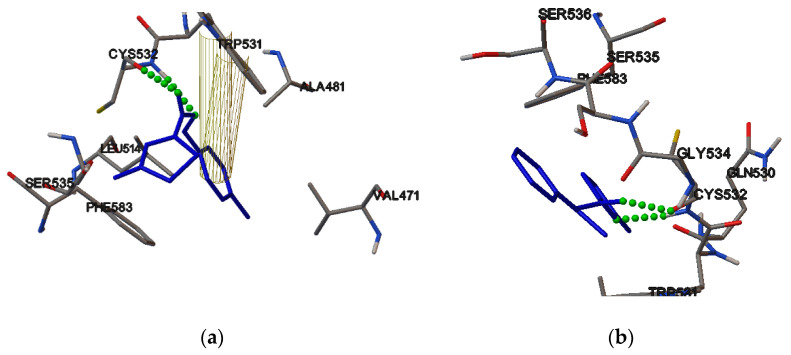
Interactions of **DKI21** (**a**) and **DKI24** (**b**) with Kinase domain.

The interactions of these two molecules with the Kinase domain are shown in Figure 10. Specifically, the **DKI21** forms two hydrogen bonds with the CYS532 amino acid and two pi–pi interactions with TRP531. In addition, **DKI24** forms two hydrogen bonds with the CYS532 amino acid. Both of the compounds bind favorably to the active center of the enzyme.

**Figure 11:**
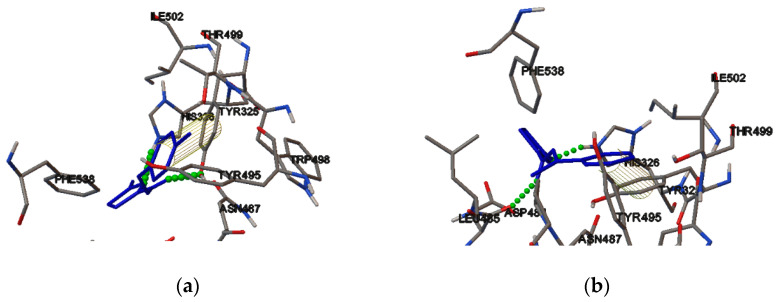
Interactions of **DKI21** (**a**) and **DKI24** (**b**) with Phosphodiesterase 4D.

The interactions of these two molecules with Phosphodiesterase 4D are shown in Figure 11. Specifically, the **DKI21** forms two hydrogen bonds with TYR325 and HIS326 amino acids and one pi–pi interaction with TYR325. In addition, the **DKI24** forms two hydrogen bonds with HIS326 and ASP484 amino acids and one pi–pi interaction with TYR326 amino acid. Both of the enzymes bind favorably to the active center of the enzyme.

**Figure 12:**
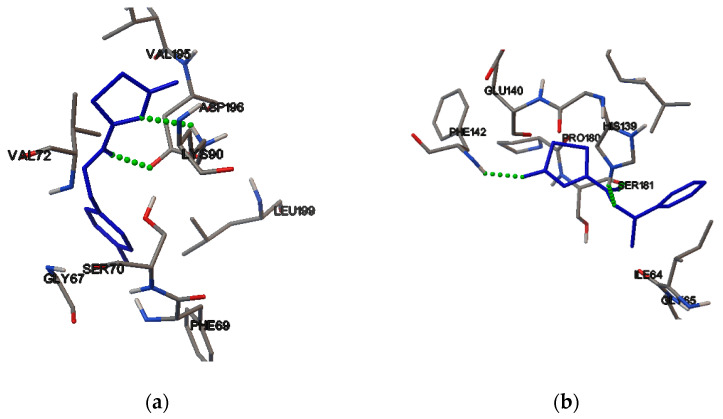
Interactions of **DKI21** (**a**) and **DKI24** (**b**) with Cell division cycle 7-related protein kinase/Activator of S phase kinase.

The interactions of these two molecules with the Cell division cycle 7-related protein kinase/Activator of S phase kinase are shown in Figure 12. Specifically, the **DKI21** forms two hydrogen bonds with the ASP196 and LYS90 amino acids. In addition, the **DKI24** forms two hydrogen bonds with the PHE142 and SER181 amino acids. Both of the compounds bind favorably to the active center of the enzyme.

**Figure 13:**
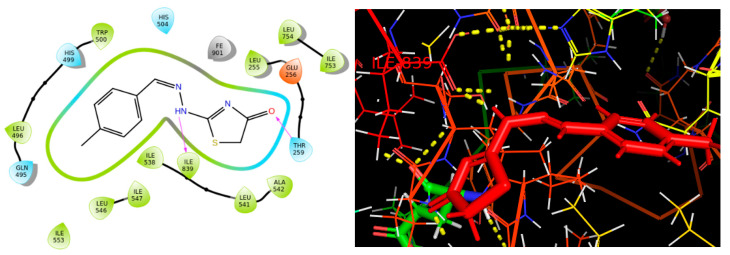
Interactions of **DKI21** with LOX-1.

Induced Fit docking was applied, in order to find how strong these two compounds bind to the active center of LOX-1. The **DKI21** binds favorably to the active site of LOX-1 with a binding score of ΔG= −7.52 kcal/mol. Specifically, the **DKI21** forms two hydrogen bonds with the ILE839 and THR259 amino acids (Figure 13).

**Figure 14:**
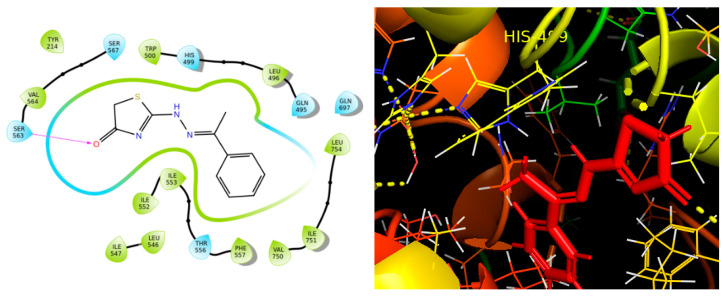
Interactions of **DKI24** with LOX-1.

In addition, **DKI24** binds favorably to the active site of LOX-1 with a binding energy of −7.54 kcal/mol. Specifically, it forms one hydrogen bond with the SER563 amino acid (Figure 14).

### 2.5. Results of the Pharmacokinetics and Toxicity Properties of the Two Compounds

Both of the compounds obey the Lipinski’s Rule of Five [24] and Veber’s Rule [25], because they have less than seven rotable bonds. Compound **DKI21** is more soluble than **DKI24** (see Table 3).

According to preADMET, the BBB [26] value is less than one. As a result, both of the compounds are classified as inactive in the central nervous system (CNS) (Table 4). The values for human intestinal absorption is high for both of the compounds, and this signifies that these compounds might be better absorbed from the intestinal tract on oral administration. Both of the compounds are not inhibitors of the CP isoenzymes, and therefore are not toxic (Table 4).

According to pkCSm (Table 5), both of the compounds have been predicted to be hepatotoxic. Compound **DKI24** has negative AMES [27] toxicity and, as a result, it is not mutagenic, in contrast to **DKI21**.

## 3. Materials and Methods

### 3.1. Synthesis

The reagents were purchased with the highest commercial quality from Aldrich, Acros, and Fluka and were used without further purification. The reactions were monitored by thin-layer chromatography (TLC), carried out on 0.25 mm silica gel plates (E. Merck silica gel 60F254), and components were visualized by UV light absorbance. Purification of compounds by column chromatography was carried out on silica gel (Merck, 70−230 mesh) and the indicated solvents. The 1H and 13C NMR spectra were recorded on Bruker 400 and 500 MHz Avance spectrometers. The 1H and 13C spectra are referenced according to the residual peak of the solvent, based on the literature data. The 13C NMR spectra are fully proton-decoupled. The electrospray ionization (ESI) mass spectral analyses were performed on a mass spectrometer, MSQ Surveyor, Finnigan, using direct sample injection. The negative or positive ion ESI spectra were acquired by adjusting the needle and cone voltages accordingly.

Synthesis: to a stirred solution of thiosemicarbazone (1 mmol) in methanol, sodium acetate (2 mmol) was added, followed by methyl-3-chloroacetate (1 mmol) and the mixture was refluxed for 2 h. A new portion of methyl-3-chloroacetate (1 mmol) was added every two hours, (2×). The reaction mixture was left to stand at room temperature, the solid was filtered, washed with methanol, to give the **DKI21** as an off-white solid in 72% yield.

Following the same procedure, the **DKI24** was obtained as an off-white solid in 77% yield.

### 3.2. Structure Assignment

The two molecules under study were structurally identified using 400 and 500 MHz spectrometers (Bruker Avance Spectrometer, Billerica, MA, USA), installed in the National and Kapodistrian University of Athens using 1D and 2D homonuclear and heteronuclear experiments. Various mixing times (d8) for 2D-NOESY were applied (0.15, 0.30, 0.5, and 0.8). The pulse sequences were obtained from the library of the spectrometer. The spectra were processed and analyzed using the MestreNova (Santiago de Compostela, Spain) and TopSpin softwares.

### 3.3. Conformational Analysis

The onformational analysis was performed to find the most stable conformation of these two compounds. All of the calculations were performed using B3LYP [29,30] as functional and 6-311G(d,p) [31]. This methodology is suitable for these organic compounds [32]. All of the calculations were performed using DMSO as a solvent, so the experimental results would be similar, and employing the polarizable continuum model (PCM) [33]. All of the conformers were fully optimized and their frequencies were calculated. Finally, all of the theoretical calculations were compared with the experimental ones. All of the calculations were performed with Gaussian 16 [34].

### 3.4. Reaction Mechanism

The mechanism of the reaction was calculated to find pit which intermediate will decide to go and which compound is most favorable.

### 3.5. Molecular Binding

AutoDock [35] software was used for the molecular binding [36] calculations and, more specifically, the Lamarckian Genetic algorithm. The crystal structures of the proteins were used by the online database “Protein Data Bank—PDB” and downloaded directly to the AutoDock program for study. The compounds used as ligands were designed with the help of the ChemOffice program, and, using the same program, their energy was minimized with an MM2 force field.

### 3.6. Induced Fit Docking

In the next stage, **DKI21** and **DKI24** were investigated for their potent binding to major targets revealed from the Swiss Target tool (http://www.swisstargetprediction.ch/, accessed on 25 June 2022) and bibliography [37,38], through molecular docking calculations. The crystal structures used for the *in silico* studies carried the following PDB IDs: *5T5V* [39]. The protein preparation wizard, a module available in Schrödinger Suites, was used to prepare the crystal structure for the *in silico* calculations. Since LOX is a metalloprotein, it has been considered that there are several computational challenges that need to be addressed. In order to take account of the quantum effects associated with the presence of a Fe^3+^ cation in LOX’s active site, we used the “create zero-order bonds to metals” module of the Schrödinger’s Maestro molecular modeling platform. This module breaks the existing bonds to metals—since they cannot be examined through the over-simplified model of a spring attached to a hard sphere—and adds new zero-order bonds between the metals and nearby atoms and corrects their formal charges accordingly, to constrain the X-ray-acquired coordination geometry.

Both of the compounds were sketched in the Schrodinger’s Maestro [40] molecular modeling platform, and it was initially minimized using Macromodel [41] and DFT calculations. LigPrep was used to prepare the 3D models, restricted to the specific stereochemistry of each molecule. During the ligand preparation, the “add metal binding states” option of the Epik module of LigPrep was chosen, in order to create ligand binding states that are suitable for metal binding that would have been rejected otherwise, due to high energy state penalties. The geometries were optimized with MacroModel in order to relax the structures, while the chiral centers retained the proper chiralities. The force field used for minimization was OPLS2005 [42]. All of the the compounds were subjected to proper treatment of their protonation states at physiological pH (~7.4). Hammett and Taft methods were implemented, in conjunction with an ionization tool to generate chemically sensible 3D models. The three-dimensional ligands’ structures were further minimized, more rigorously, by MacroModel, with water as the solvent and OPLS2005 as the force field, using a conjugate gradient (CG) method with a threshold of 0.01 kcal/mol. The minimized structure was further used as input to a mixed-torsional/low-sampling conformational search forced to keep the input chiralities. The conformational search generated a number of conformers for each molecule under study and the conformers were energetically ranked. The most favored conformation was used as input for the following docking calculations.

The docking calculations were performed to reveal the possible binding of compounds to LOX-1. The calculations were performed with the Induced Fit Docking (IFD) method. The ligand was docked in the 10 energetically favored conformations generated by Macromodel. The protein preparation constrained refinement was applied in the Glide docking stage. Trimming the side chains automatically (based on B—factor) and a Prime refinement of the protein side chains were applied and the docking process was accomplished by Glide/XP. Finally, the binding energy was calculated. The active site was described, using a dielectric constant of 80 and all of the crystallographic waters of the active site were preserved.

### 3.7. ADMET Calculations

Both of the compounds were sketched in ChemDraw, in order to find their SMILES. After their SMILES were found, they were imported in SwissADME [43], pkCSM [28], and pro-TOX to find their pharmacological and toxicological results. This procedure is very important for computational drug design, because the vast majority of drugs that do not reach the market are either due to limited effectiveness or due to side effects.

## 4. Conclusions

This study focuses on structure assignment and conformational analysis of two promising bioactive thiosemicarbazone adducts, **DKI21** and **DKI24**, using a combination of NMR spectroscopy and computational studies (QM methods). The NMR experiments were performed, using a combination of 2D-ROESY and 2D-NOESY at different mixing times to assign unambiguously the *exo* structure of the two molecules under study. The calculation of the mechanistic pathway of the reaction proves that the molecules obtain *exo* configuration. *In silico* experiments were performed to find some possible biological targets for the two molecules under study. The results showed that the compounds bind favorably to the revealed enzyme targets, using Swissadme software. Both of the derivatives obey Lipinski’s Rule of Five and Veber’s Rule. It appears that both of the molecules are predicted not to have toxic properties and to be bioactive for various biological targets. Thus, these molecules can be promising leads for these targets. In addition, the derivatives of these compounds can be synthesized by medicinal chemists to optimize their biological targeting.

## Figures and Tables

**Table 1:** Assignment of the experimental ^1^H-NMR spectra of **DKI21** (top) and **DKI24** (below) in DMSO-d6.

Position	^1^H	COSY	NOESY	HSQC	HMBC	^13^C
1	2.35	-	H-3	C-1	C-2. C-3	21.51
2	-	-	-	-	H-1/H-3	141.03
3	7.26	H-4	H-1/H-4	C-3	C-2/C-5	129.90
4	7.66	H-3	H-3/H-6	C-4	C-5/C-3/C-6	128.10
5	-	-	-	-	H-3/H-6/H-4	131.98
6	8.36		H-4	C-6	H-5/H-4	156.68
7						
8						
9	-	-	-	-	C-11/C-12/H-8	165.20
10						
11	-	-	-	-	H-12	174.62
12	3.89	-	-	C-12	C-11/C-9	33.45
Position	^1^H	COSY	NOESY	HSQC	HMBC	^13^C
1	7.47	H-2	H-2	C-1	C-2. C-3	121.87
2	7.47	H-1/H-3	H-1/H-3	C-2	H-1/H-3	121.87
3	7.86	H-2	H-2/H-6	C-3	C-2/C-5/C-4	126.84
4	-	-	-	-	H-3/H-2/H-6	138.22
5	-	-	-	-	H-3/H-6/H-4	160.83
6	2.37	-	H-3	C-6	H-5/H-4	15.04
7						
8						
9	-	-	-	-	H-12	164.53
10						
11	-	-	-	-	H-12	174.42
12	3.85	-	-	C-12	C-11/C-9	33.26

**Table 2:** Binding energies BE (kcal/mol) and inhibition constants between the compounds and macromolecules.

	Binding Energy	Inhibition Constant
2VRM (MAO B)		
**DKI21**	−7.64 ± 0.5	2.52 ± 0.5 μΜ/3.13 ± 0.5 μΜ
**DKI24**	−7.55 ± 0.5	2.93 ± 0.5 μΜ
6ME2 (Melatonin receptor MT1)		
**DKI21**	−6.81 ± 0.5	10.17 ± 0.5 μΜ
**DKI24**	−7.38 ± 0.5	3.92 ± 0.5 μΜ
6MHM (Human acid ceramidase)		
**DKI21**	−7.10 ± 0.5	6.29 ± 0.5 μΜ
**DKI24**	−7.07 ± 0.5	6.58 ± 0.5 μΜ
5HIE (Kinase domain)		
**DKI21**	−6.41 ± 0.5	19.9 ± 0.5 μΜ
**DKI24**	−6.36 ± 0.5	21.89 ± 0.5 μΜ
3G4L(Phosphodiesterase 4D)		
**DKI21**	−7.1 ± 0.5	6.3 ± 0.5 μΜ
**DKI24**	−7.76 ± 0.5	2.05 ± 0.5 μΜ
2RJQ (ADAMTS5)		
**DKI21**	−7.65 ± 0.5	2.48 ± 0.5 μΜ
**DKI24**	−7.7 ± 0.5	4.47 ± 0.5 μΜ
CDC7/DBF4 (Cell division cycle 7-related protein kinase/Activator of S phase kinase) (4F9C)		
**DKI21**	−6.07 ± 0.5	35.5 ± 0.5 μΜ
**DKI24**	−6.20 ± 0.5	28.43 ± 0.5 μΜ

**Table 3:** The physicochemical parameters for compounds **DKI21** and **DKI24**.

Properties	Compound DK121	Compound DΚΙ24
Molecular Weight	233.296	233.296
LogP	1.54792	1.6296
Rotable bonds	2	2
Hydrogen Bond Acceptors	4	4
Hydrogen Bond Donors	1	1
Surface Area	97.846 (Å^2^)	97.846 (Å^2^)
Water solubility	−2.855 (logmolL^−1^)	−2.702 (logmolL^−1^)

**Table 4:** The ADME results of **DKI21** and **DKI24** according to preADMET.

	Compound DKI21	Compound DKI24
BBB	0.121871	0.370766
Buffer_solubility_mg_L	101.478	11192.1
Caco2	8.61083	7.52339
CYP_2C19_inhibition	Non	Non
CYP_2C9_inhibition	Non	Non
CYP_2D6_inhibition	Non	Non
CYP_2D6_substrate	Non	Non
CYP_3A4_inhibition	Non	Non
CYP_3A4_substrate	Non	Weakly
HIA	96.096918	96.096626
MDCK	23.4668	22.523
Pgp_inhibition	Non	Non
Plasma_Protein_Binding	73.336958	70.164297
Pure_water_solubility_mg_L	12.6628	20.7683
Skin_Permeability	−3.1538	−3.40889

**Table 5:** Toxicity results of **DKI21** and **DKI24** according to pKCSm [28].

Properties	Compound DK121	Compound DKI24
Toxicity		
AMES toxicity	Yes	No
Max. tolerated dose (human)	0.399 (log mg/kg/day)	0.269 (log mg/kg/day)
Herg I inhibitor	No	No
Herg II inhibitor	No	No
Oral Rat Acute Toxicity (LD50)	2.699 (mol/kg)	2.878 (mol/kg)
Oral Rat Chronic Toxicity	1.417 (log mg/kg_bw/day)	1.386 (log mg/kg_bw/day)
Hepatotoxicity	Yes	Yes
Skin Sensitization	Yes	Yes

## Data Availability

The data presented in this paper are available in the Supplementary Materials.

## Supplementary Materials

The following supporting information can be downloaded at https://www.mdpi.com/article/10.3390/molecules27144548/s1, Figure S1: ^1^H-NMR spectra. The spectra were recorded in DMSO-d6 on a Bruker AC 400 MHz spectrometer at 25 °C; Figure S2: 2D-NOESY-NMR spectra. The spectra were recorded in DMSO-d6 on a Bruker AC 400 MHz spectrometer at 25 °C; Figure S3: ^13^C-NMR spectra. The spectra were recorded in DMSO-d6 on a Bruker AC 400 MHz spectrometer at 25 °C; Figure S4: 2D-HSQC-NMR spectra. The spectra were recorded in DMSO-d6 on a Bruker AC 400 MHz spectrometer at 25 °C; Figure S5: 2D-HMBC-NMR spectra. The spectra were recorded in DMSO-d6 on a Bruker AC 400 MHz spectrometer at 25 °C; Figure S6: ^1^H-NMR spectra. The spectra were recorded in DMSO-d6 on a Bruker AC 400 MHz spectrometer at 25 °C; Figure S7: 2D-NOESY-NMR spectra. The spectra were recorded in DMSO-d6 on a Bruker AC 400 MHz spectrometer at 25 °C; Figure S8: ^13^C-NMR spectra. The spectra were recorded in DMSO-d6 on a Bruker AC 400MHz spectrometer at 25 °C; Figure S9: 2D-HSQC-NMR spectra. The spectra were recorded in DMSO-d6 on a Bruker AC 400 MHz spectrometer at 25 °C; Figure S10: 2D-HMBC-NMR spectra. The spectra were recorded in DMSO-d6 on a Bruker AC 400 MHz spectrometer at 25 °C; Figure S11: 2D-ROESY-NMR spectra. The spectra were recorded in DMSO-d6 on a Bruker AC 500 MHz spectrometer at 25 °C using P15 = 150.000 us; Figure S12: 2D-ROESY-NMR spectra. The spectra were recorded in DMSO-d6 on a Bruker AC 500 MHz spectrometer at 25 °C using P15 = 300.000 us; Figure S13: 2D-ROESY-NMR spectra. The spectra were recorded in DMSO-d6 on a Bruker AC 500 MHz spectrometer at 25 °C using P15 = 500.000 us; Figure S14: 2D-ROESY-NMR spectra. The spectra were recorded in DMSO-d6 on a Bruker AC 500 MHz spectrometer at 25 °C using P15 = 150.000 us; Figure S15: 2D-ROESY-NMR spectra. The spectra were recorded in DMSO-d6 on a Bruker AC 500 MHz spectrometer at 25 °C using P15 = 300.000 us; Figure S16: 2D-ROESY-NMR spectra. The spectra were recorded in DMSO-d6 on a Bruker AC 500 MHz spectrometer at 25 °C using P15 = 500.000 us; Figure S17: Overall diagram showing the identification strategy of the **DKI21** compound in DMSO; Figure S18: Overall diagram showing the identification strategy of the DKI24 compound in DMSO; Figure S19: Orbitals in HOMO (top) and LUMO (bottom) in **DKI21** *exo* conformation; Figure S20: Orbitals in HOMO (top) and LUMO (bottom) in **DKI24** *exo* conformation; Figure S21: Orbitals in HOMO (top) and LUMO (bottom) in **DKI21** *endo* conformation; Figure S22: Orbitals in HOMO (top) and LUMO (bottom) in **DKI24** *endo* conformation; Table S1: HOMO-LUMO gap, hardness, and softness of lowest in energy *endo* and *exo* conformers.

## Sample Availability

Samples of the compounds **DKI21** and **DKI24** are available from the authors.
