# Supplementary material for: Conformational Properties and Putative Bioactive Targets for Novel Thiosemicarbazone Derivatives
Source: Molecules. 2022 Jul 16;27(14):4548. doi: 10.3390/molecules27144548 (PMC9324535; doi:10.3390/molecules27144548)
Supplement: Supplementary file 1 [file molecules-27-04548-s001.zip › molecules-1806106-supplementary.pdf]

SUPPORTING INFORMATION

Nikitas Georgiou [1], Antigoni Cheilari [2], Danai Karta [1], Eleni Chontzopoulou [1], Janez Plavec [3], Demeter Tzeli [4,5], Stamatia Vassiliou [1,*] and Thomas Mavromoustakos [1,*]

[1] Laboratory of Organic Chemistry, Department of Chemistry, National and Kapodistrian University of
Athens, Panepistimioupolis Zografou, 11571 Athens, Greece;
nikitage@chem.uoa.gr (N.G.);
danaikarta@gmail.com (D.K.); elenichontzo@chem.uoa.gr (E.C.)

[2] Department of Pharmacognosy and Natural Products Chemistry, Faculty of Pharmacy, National and
Kapodistrian University of Athens, Panepistimiopolis Zografou, 15771 Athens, Greece;
cheilarianti@pharm.uoa.gr

[3] Slovenian NMR Centre, National Institute of Chemistry, SI-1001 Ljubljana, Slovenia; janez.plavec@ki.si

[4] Laboratory of Physical Chemistry, Department of Chemistry, National and Kapodistrian University of
Athens, Panepistimioupolis Zografou, 11571 Athens, Greece;
tzeli@chem.uoa.gr

[5] Theoretical and Physical Chemistry Institute, National Hellenic Research Foundation, 48 Vassileos
Constantinou Ave., 11635 Athens, Greece

* Correspondence: svassiliou@chem.uoa.gr (S.V.); tmavrom@chem.uoa.gr (T.M.)

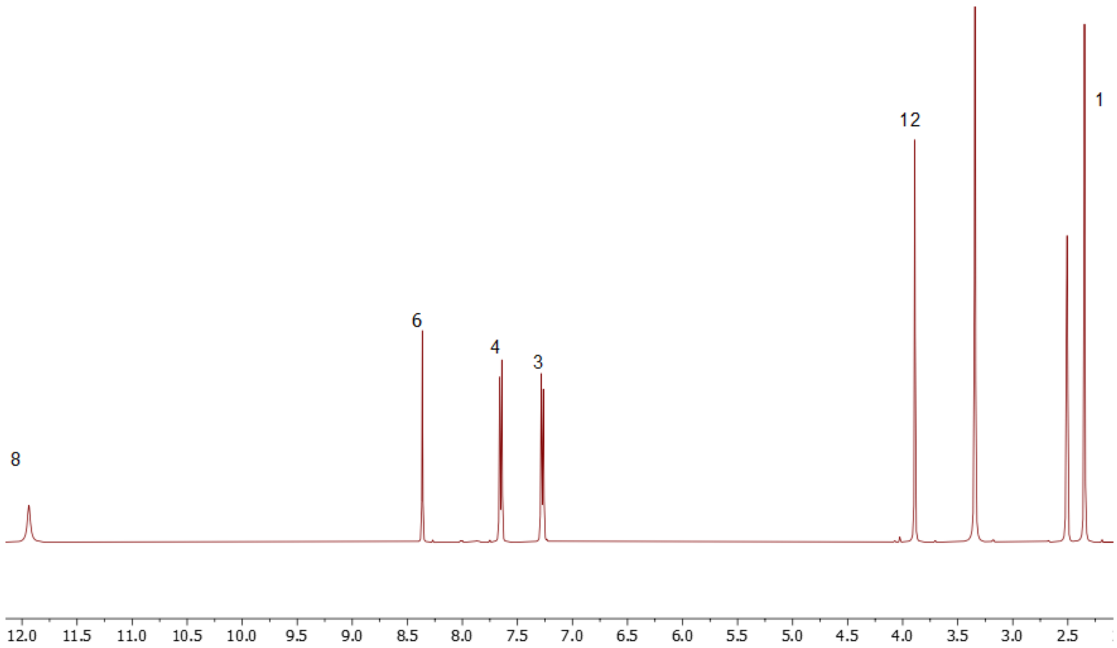

Figure S1. ^{1}H-NMR spectra. The spectra were recorded in DMSO-d6 on a Bruker AC 400MHz spectrometer at 25ºC.

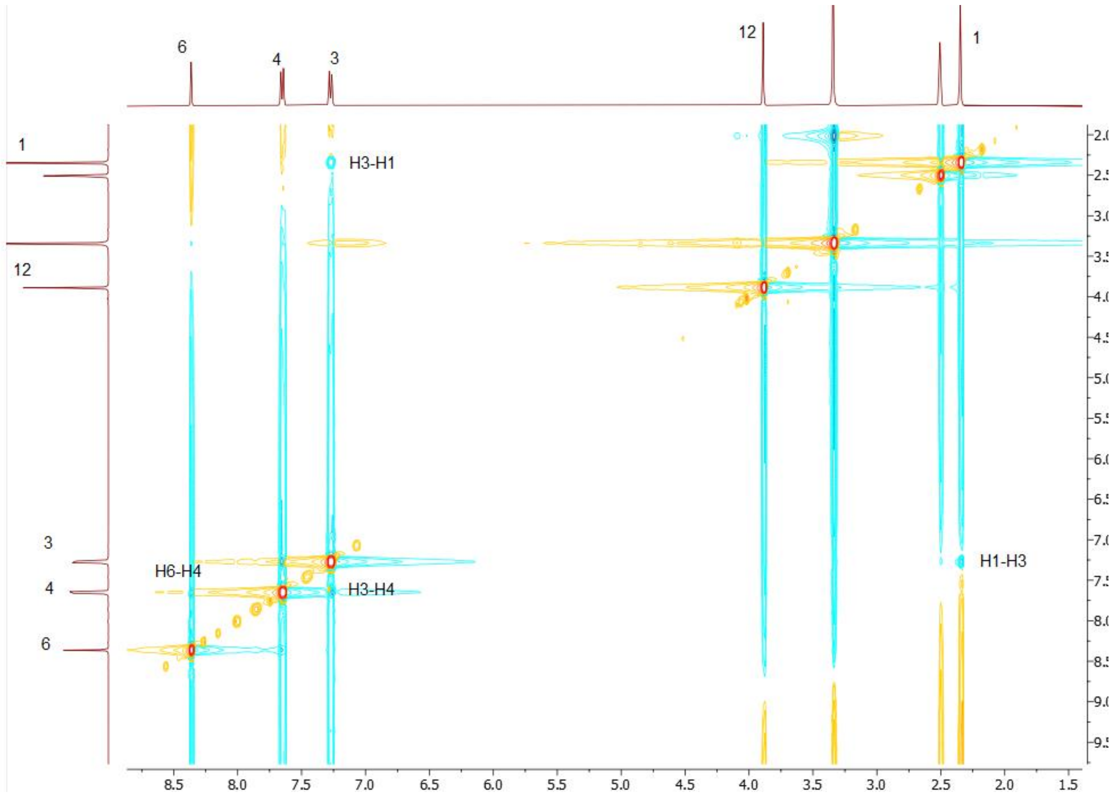

Figure S2. 2D-NOESY-NMR spectra. The spectra were recorded in DMSO-d6 on a Bruker AC 400MHz spectrometer at 25ºC.

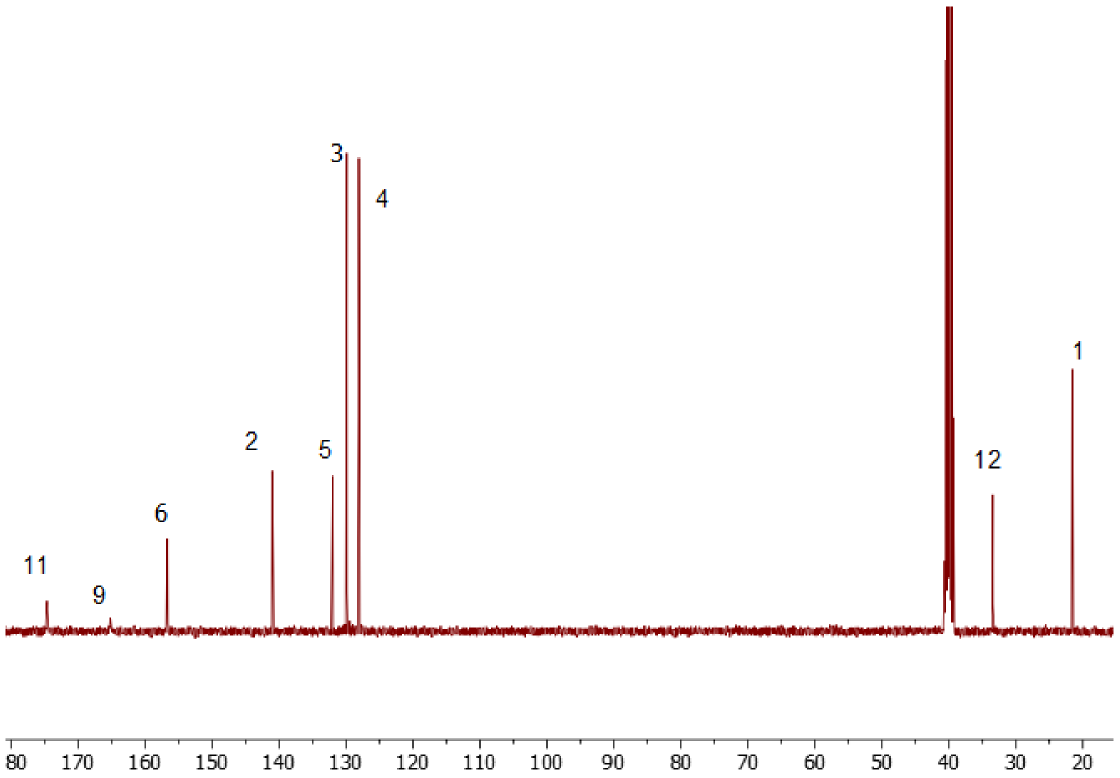

Figure S3. ^{13}C-NMR spectra. The spectra were recorded in DMSO-d6 on a Bruker AC 400MHz spectrometer at 25ºC.

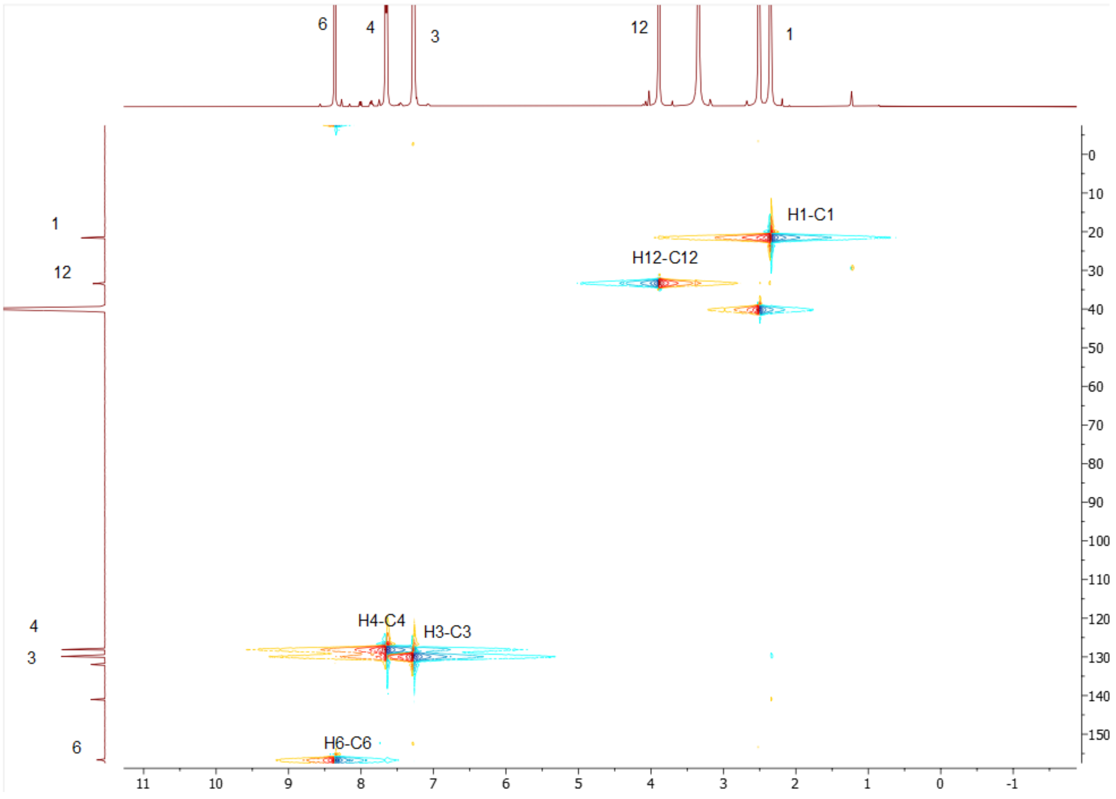

Figure S4. 2D-HSQC-NMR spectra. The spectra were recorded in DMSO-d6 on a Bruker AC 400MHz spectrometer at 25ºC.

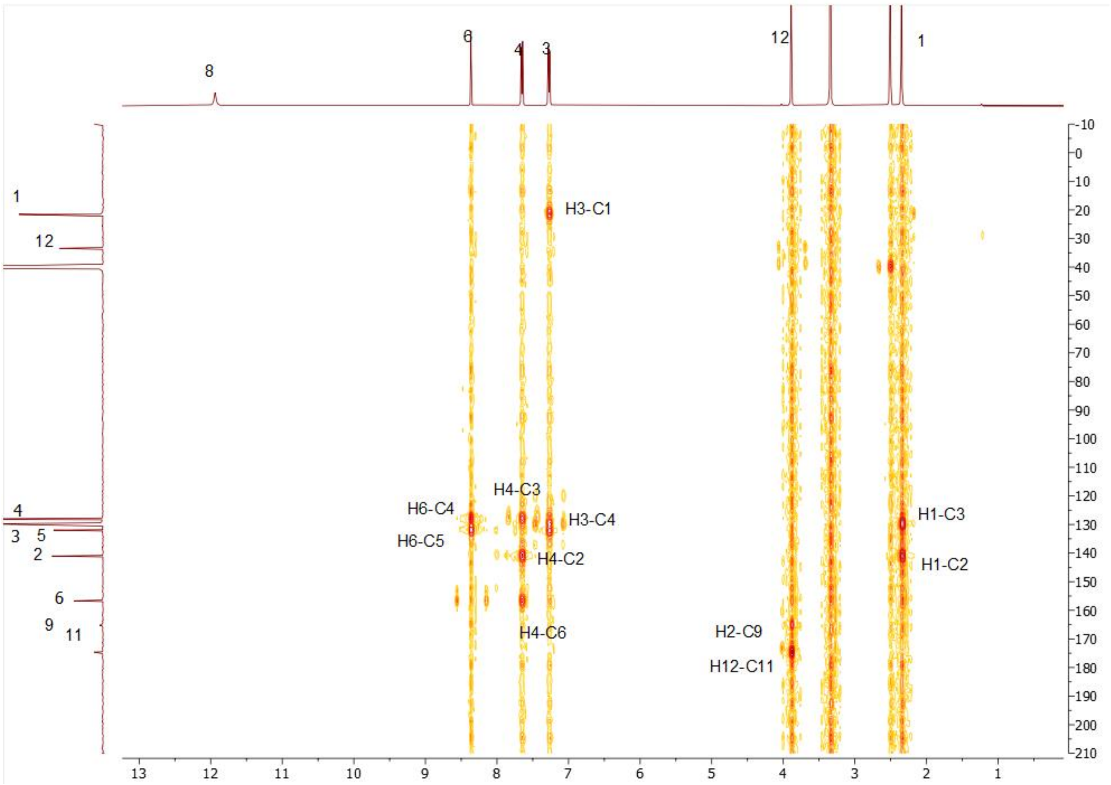

Figure S5. 2D-HMBC-NMR spectra. The spectra were recorded in DMSO-d6 on a Bruker AC 400MHz spectrometer at 25ºC.

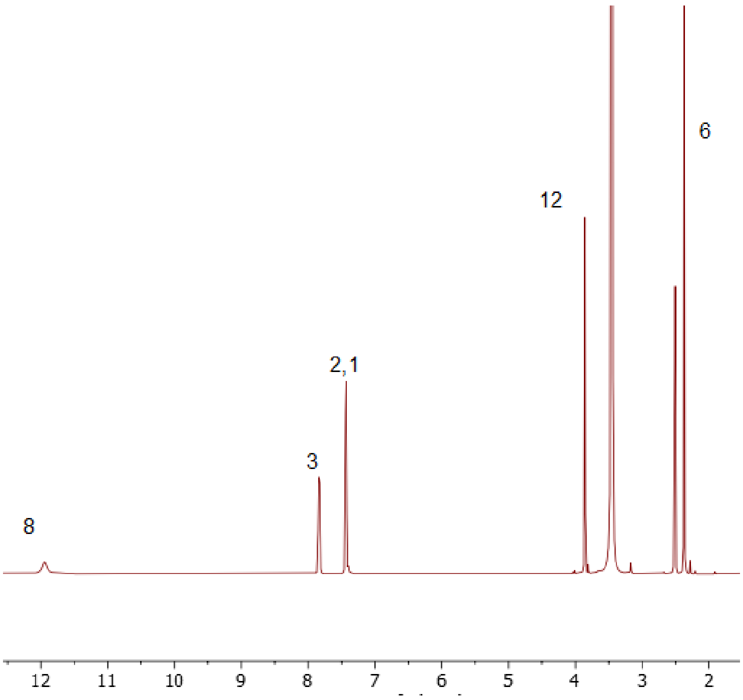

Figure S6. ^{1}H-NMR spectra. The spectra were recorded in DMSO-d6 on a Bruker AC 400MHz spectrometer at 25ºC.

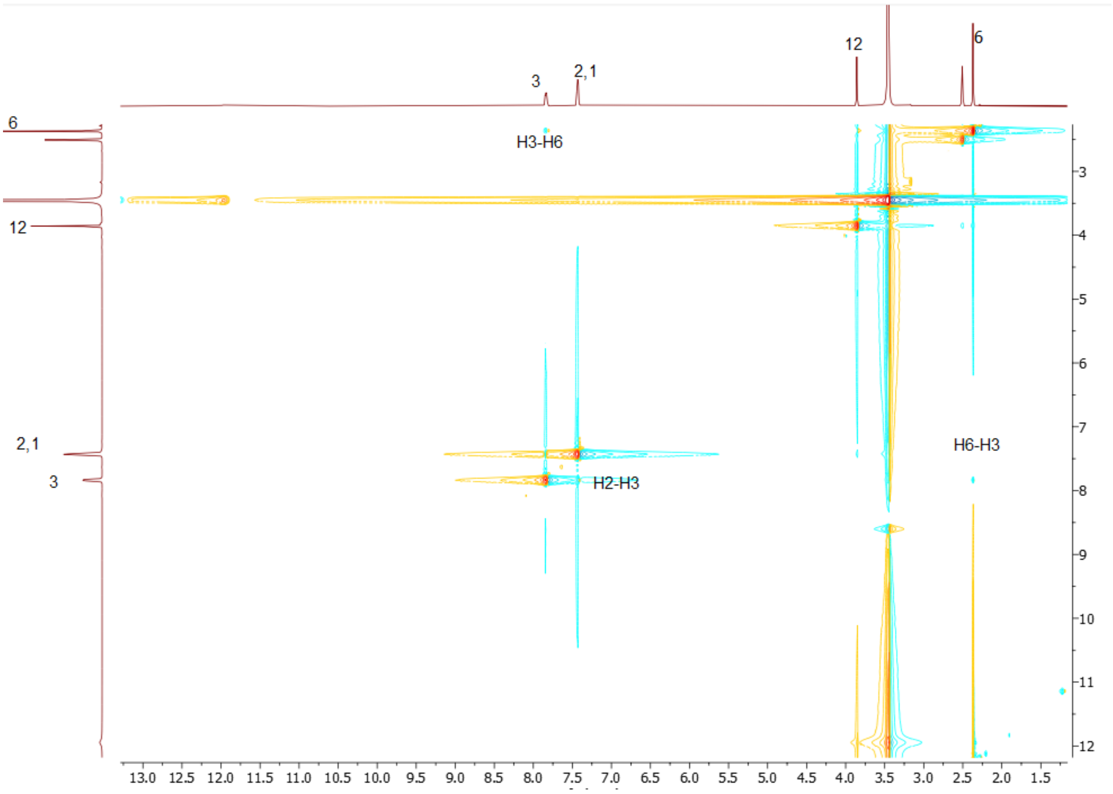

Figure S7. 2D-NOESY-NMR spectra. The spectra were recorded in DMSO-d6 on a Bruker AC 400MHz spectrometer at 25°C.

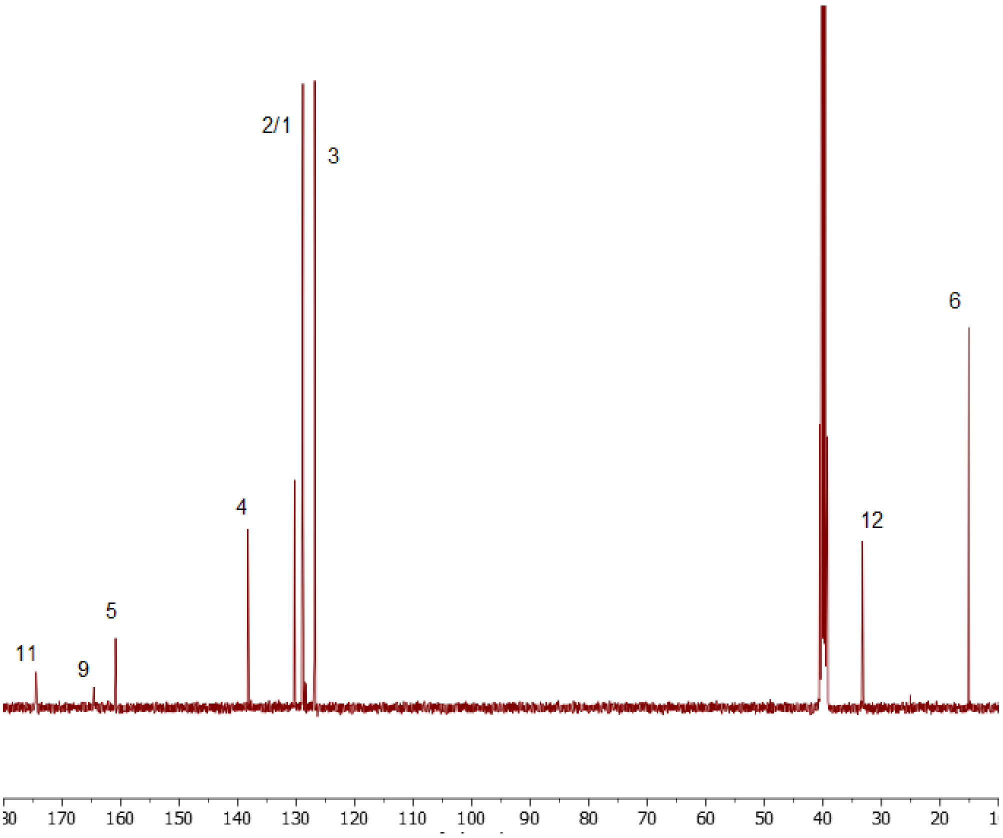

Figure S8. ^{13}C-NMR spectra. The spectra were recorded in DMSO-d6 on a Bruker AC 400MHz spectrometer at 25°C.

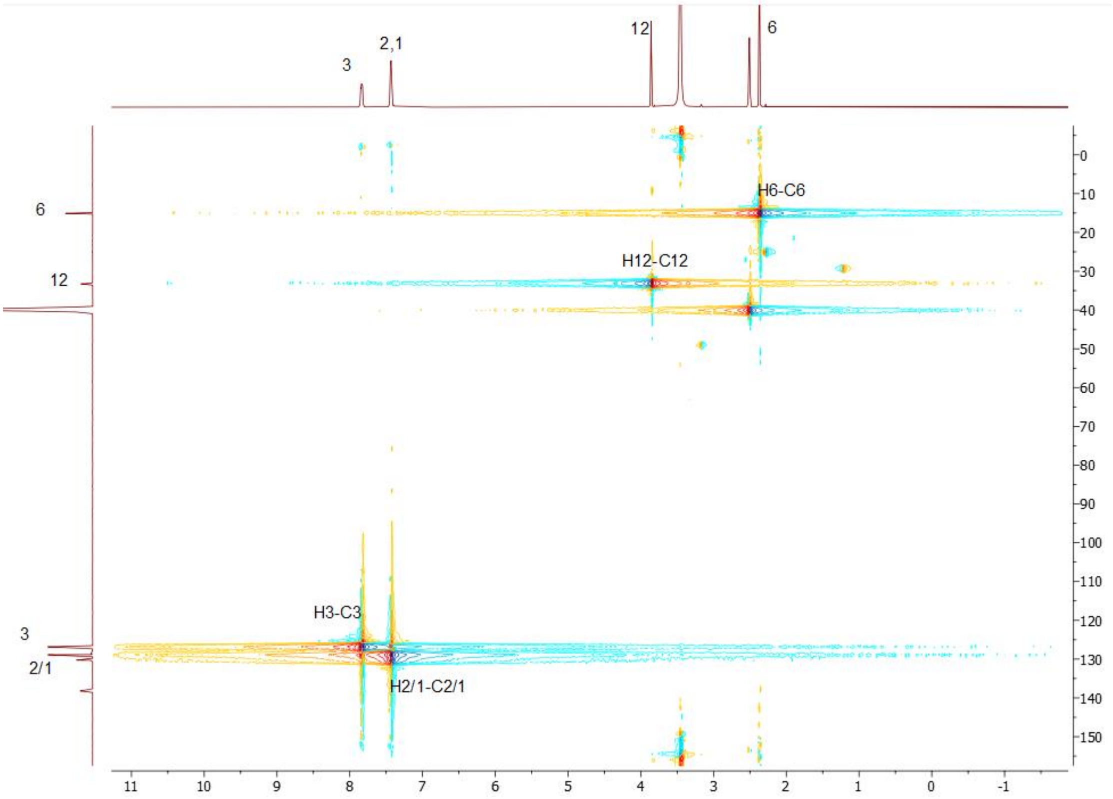

Figure S9. 2D-HSQC-NMR spectra. The spectra were recorded in DMSO-d6 on a Bruker AC 400MHz spectrometer at 25ºC.

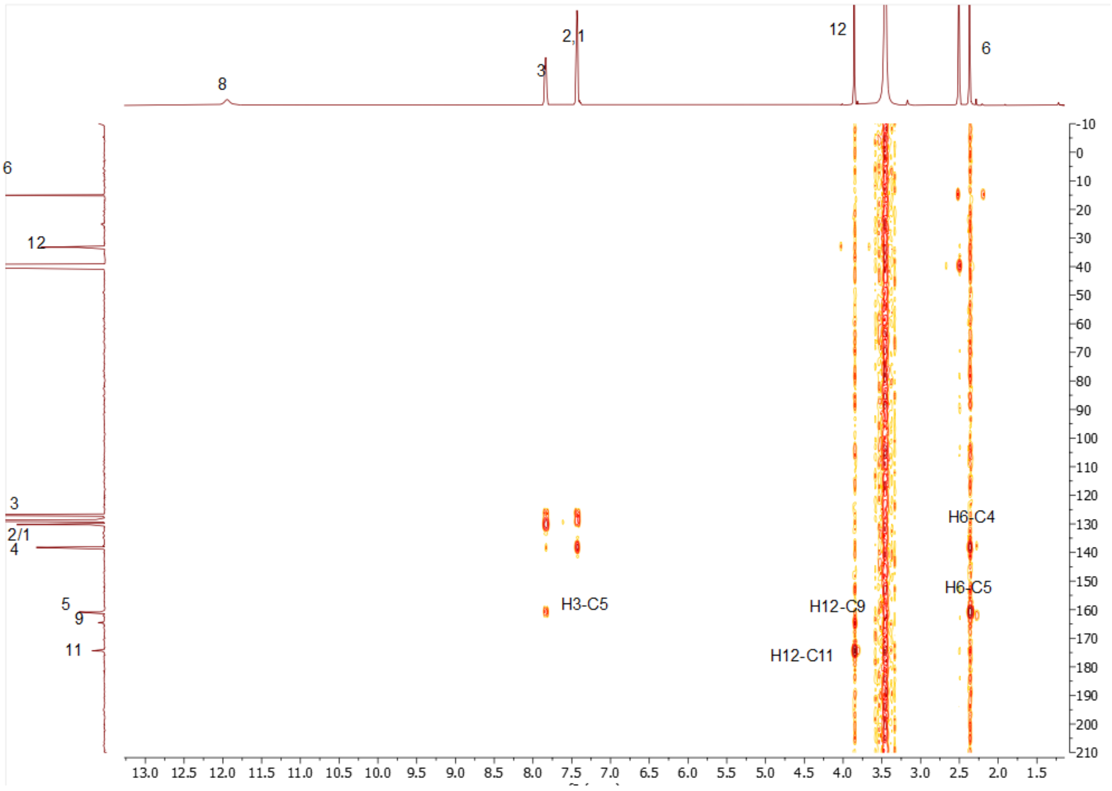

Figure S10. 2D-HMBC-NMR spectra. The spectra were recorded in DMSO-d6 on a Bruker AC 400MHz spectrometer at 25ºC.

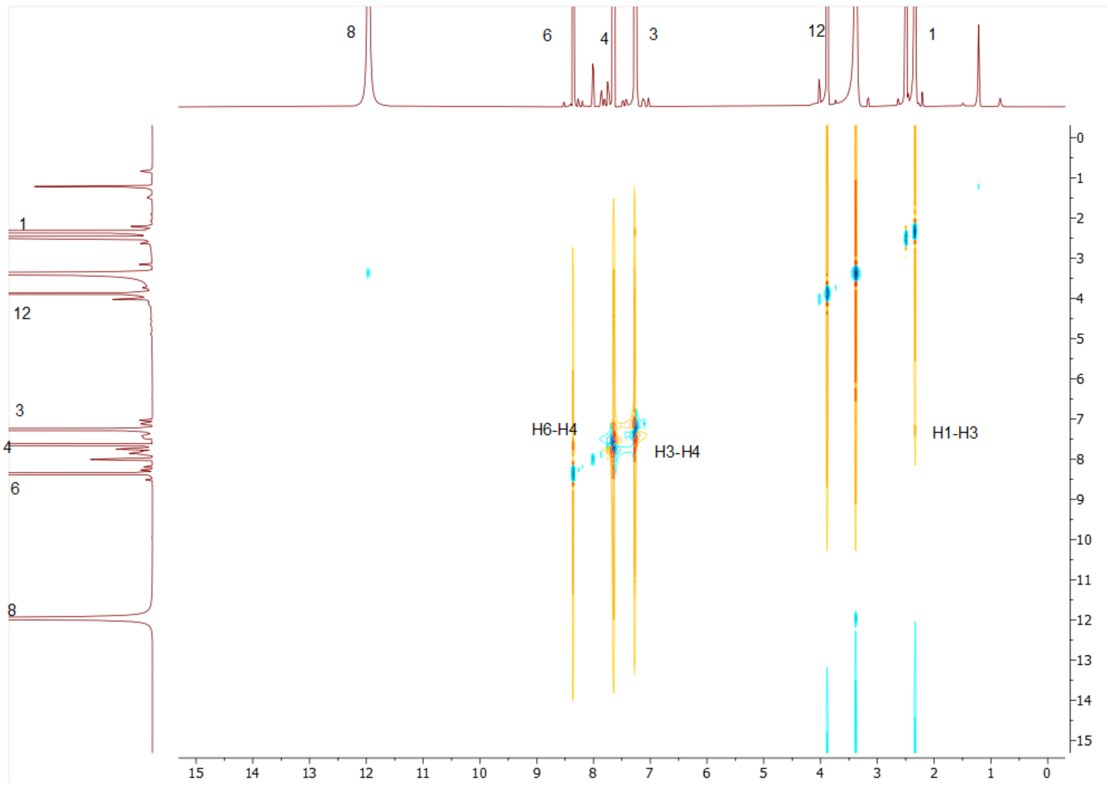

Figure S11. 2D-ROESY-NMR spectra. The spectra were recorded in DMSO-d6 on a Bruker AC 500MHz spectrometer at 25ºC using P15=150.000 us.

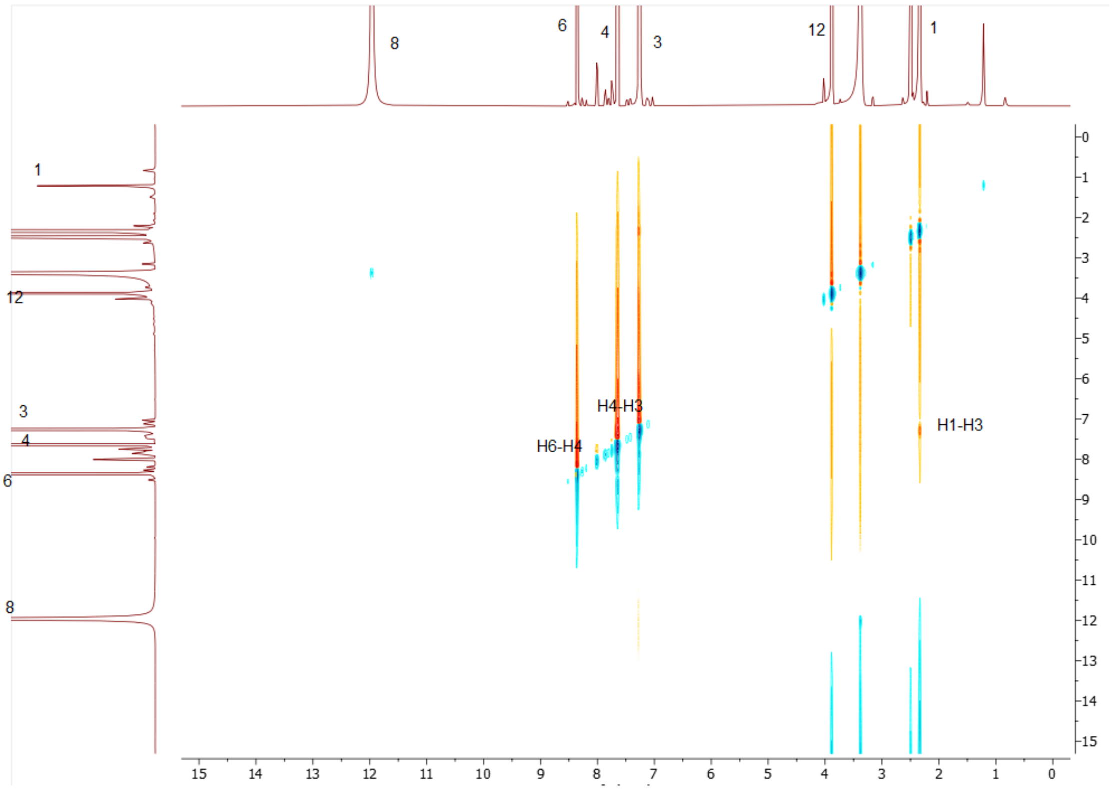

Figure S12. 2D-ROESY-NMR spectra. The spectra were recorded in DMSO-d6 on a Bruker AC 500MHz spectrometer at 25ºC using P15=300.000 us.

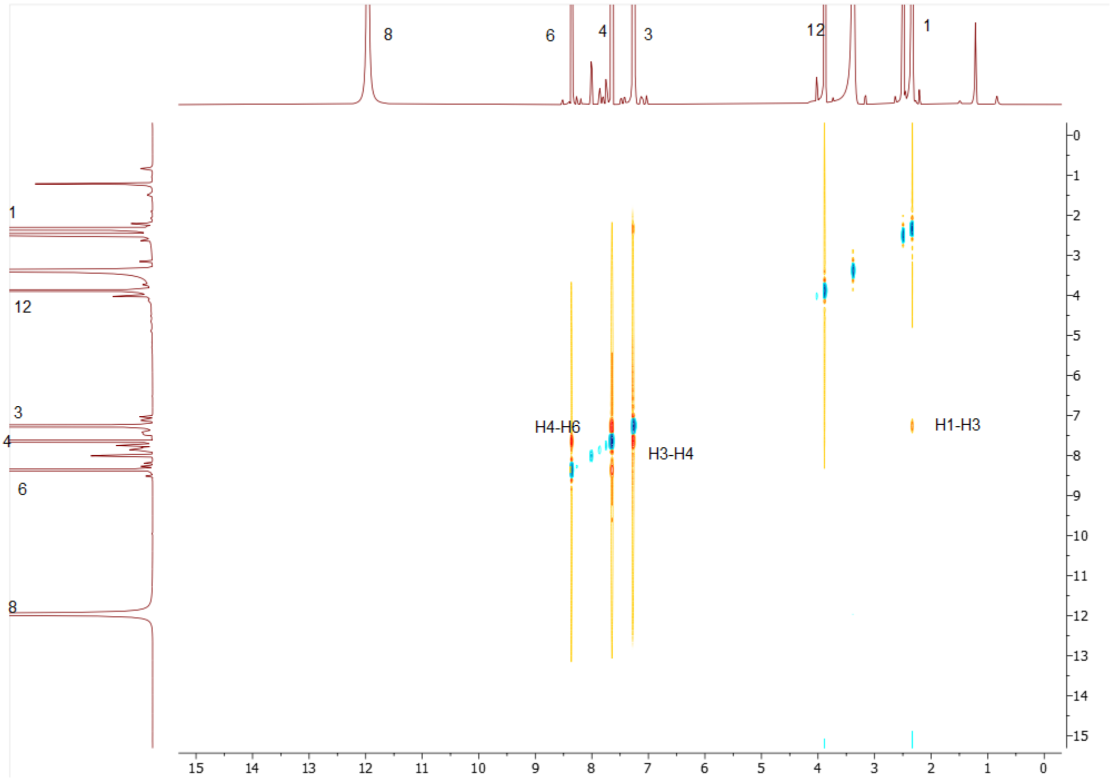

Figure S13. 2D-ROESY-NMR spectra. The spectra were recorded in DMSO-d6 on a Bruker AC 500MHz spectrometer at 25ºC using P15=500.000 us.

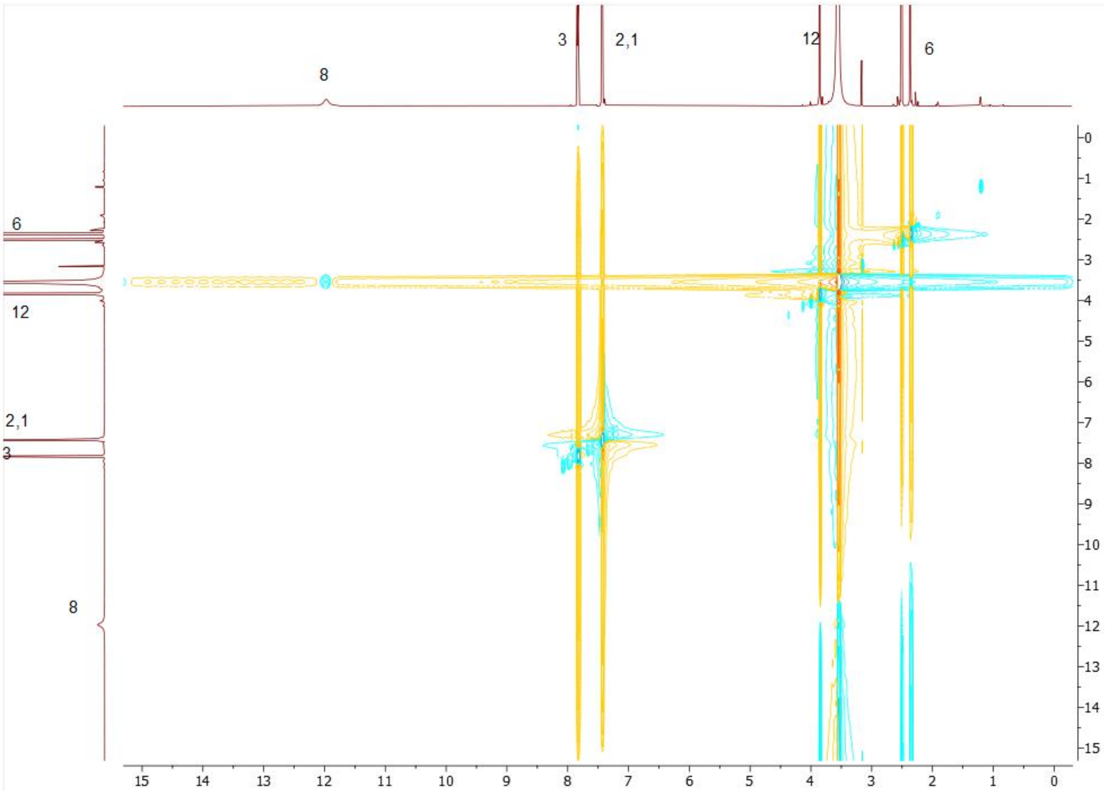

Figure S14. 2D-ROESY-NMR spectra. The spectra were recorded in DMSO-d6 on a Bruker AC 500MHz spectrometer at 25ºC using P15=150.000 us.

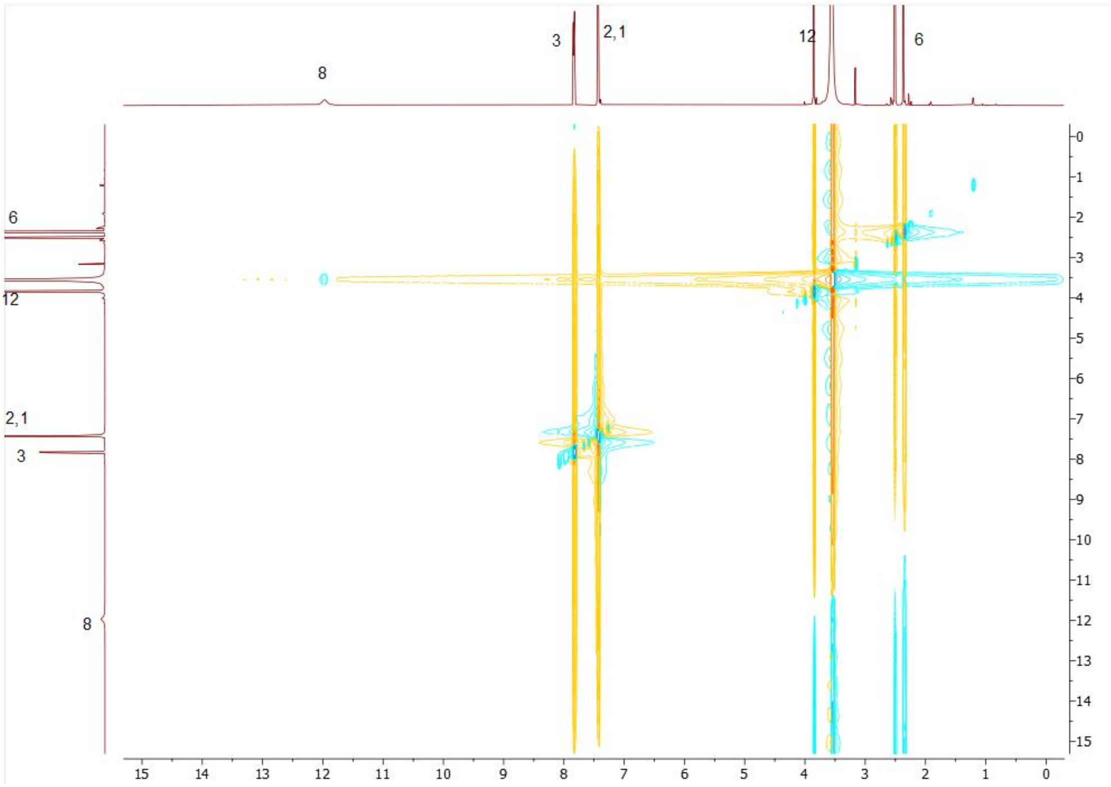

Figure S15. 2D-ROESY-NMR spectra. The spectra were recorded in DMSO-d6 on a Bruker AC 500MHz spectrometer at 25ºC using P15=300.000 us.

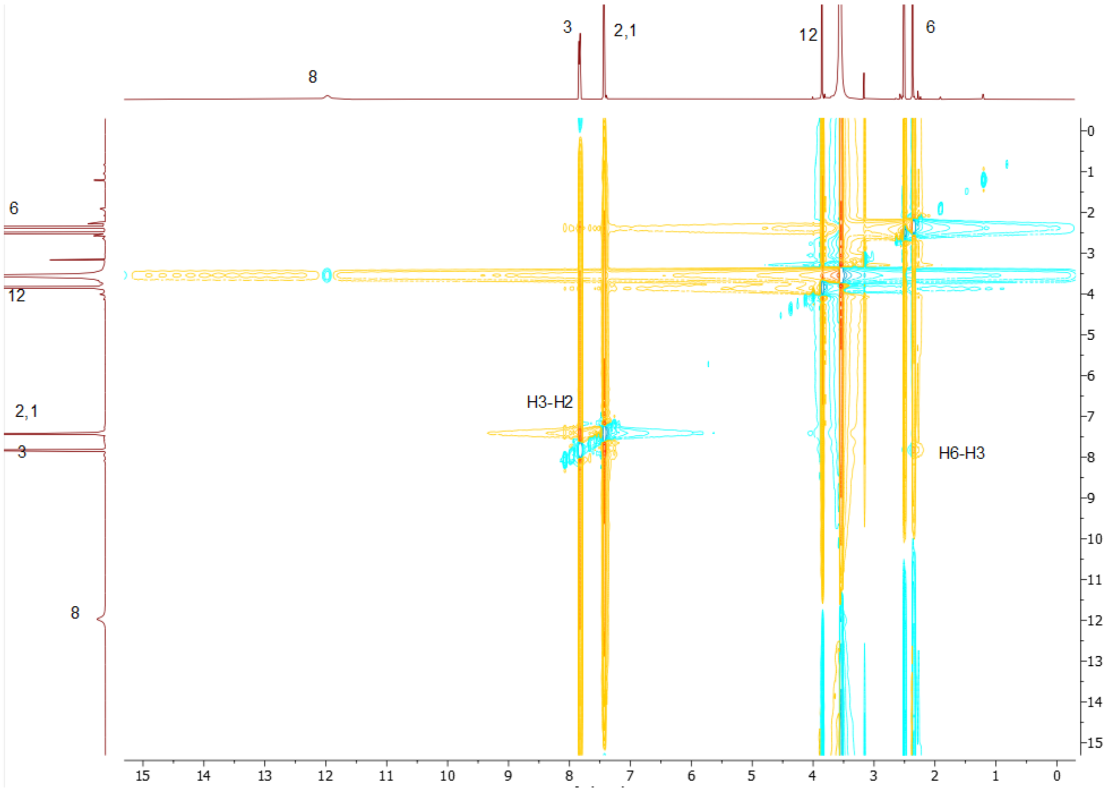

Figure S16. 2D-ROESY-NMR spectra. The spectra were recorded in DMSO-d6 on a Bruker AC 500MHz spectrometer at 25ºC using P15=500.000 us.

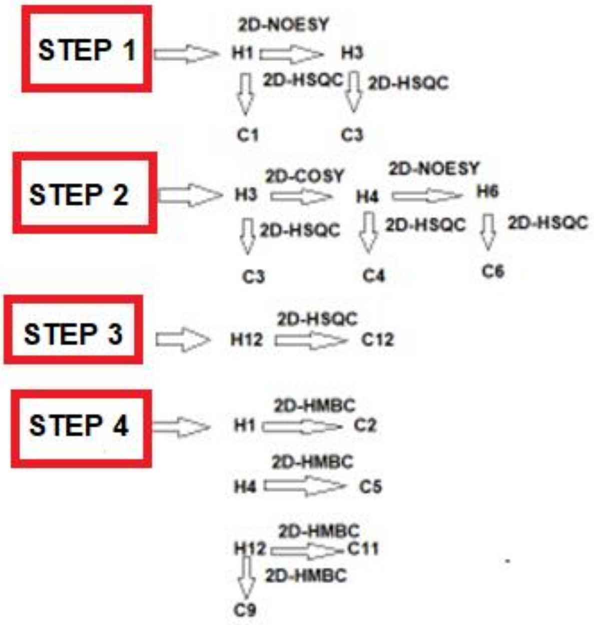

Figure S17. Overall diagram showing the identification strategy of the **DKI21** compound in DMSO.

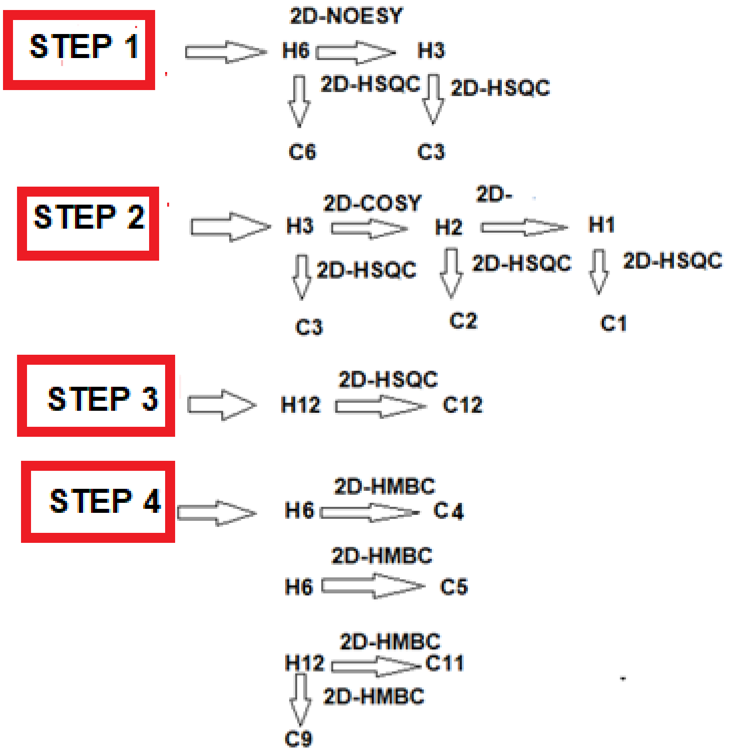

Figure S18. Overall diagram showing the identification strategy of the **DKI24** compound in DMSO.

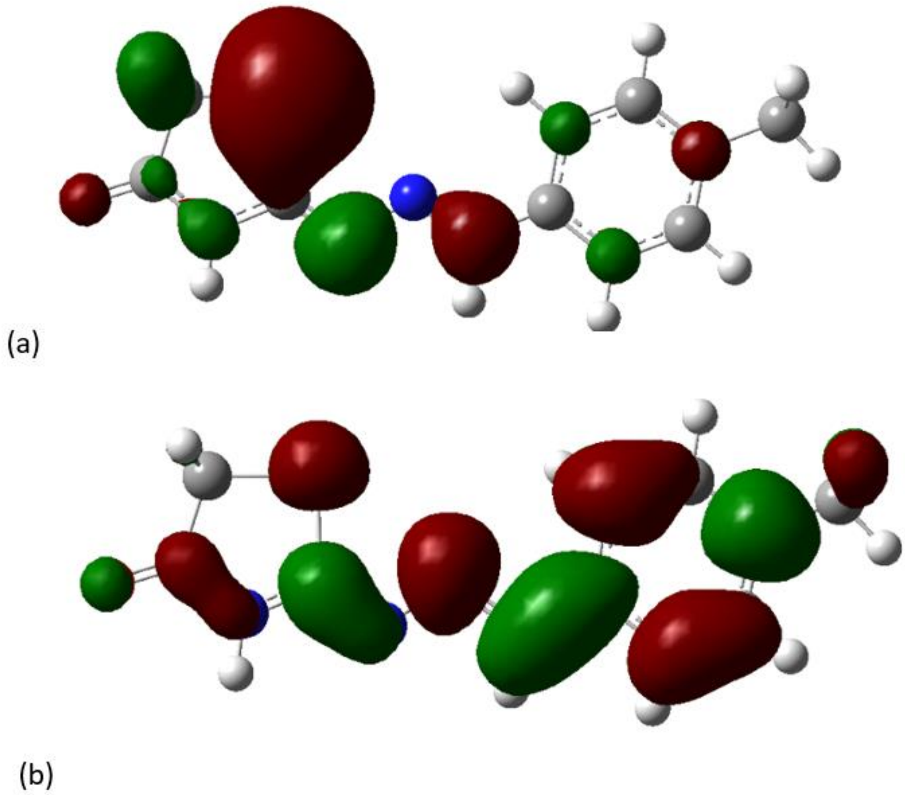

Figure S19. Orbitals in HOMO (top) and LUMO (bottom) in **DKI21** exo conformation.

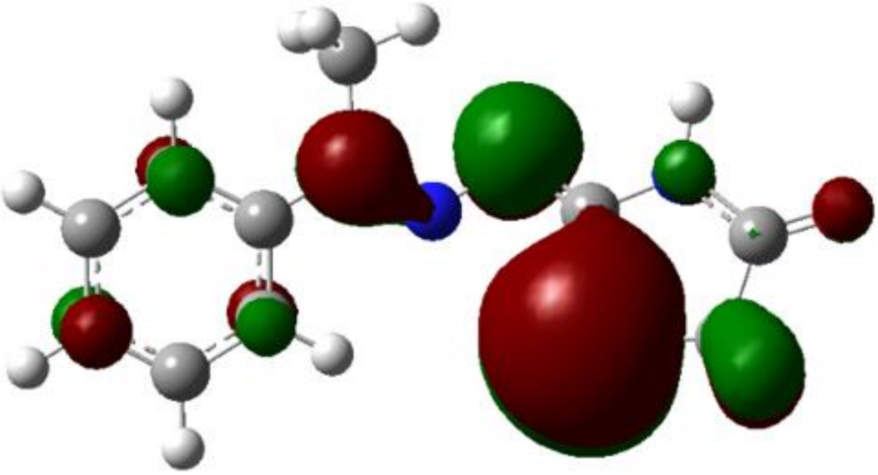

(a)

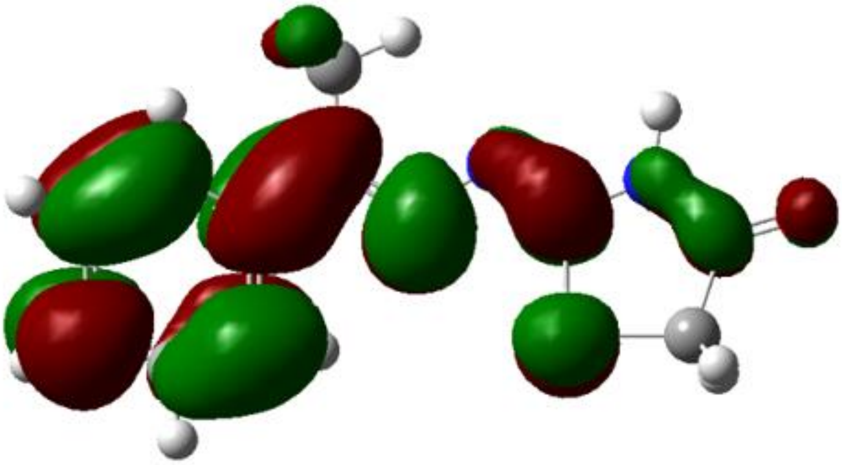

(b)

Figure S20. Orbitals in HOMO (top) and LUMO (bottom) in **DKI24** exo conformation

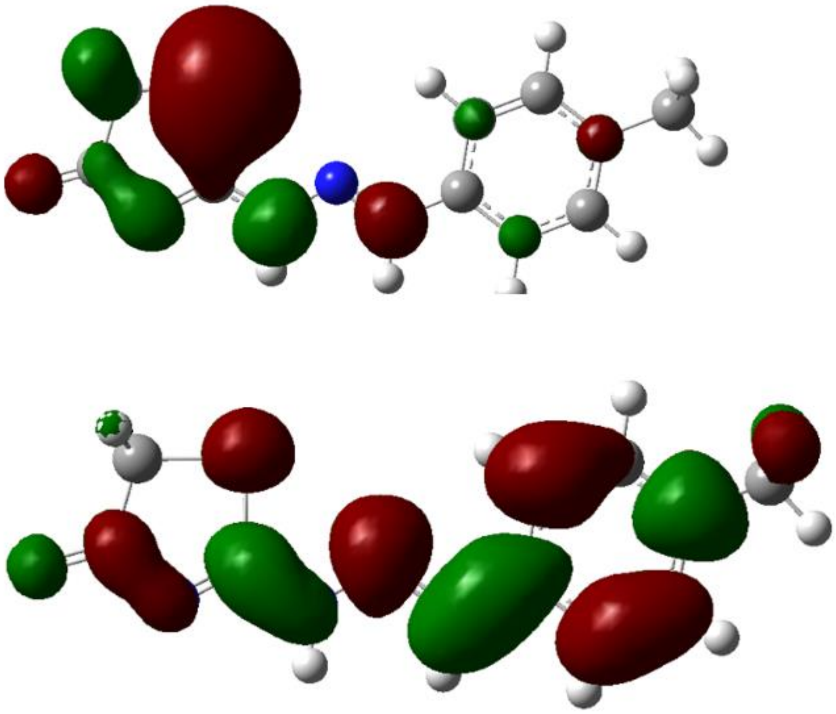

(a)

(b)

Figure S21. Orbitals in HOMO (top) and LUMO (bottom) in **DKI21** endo conformation.

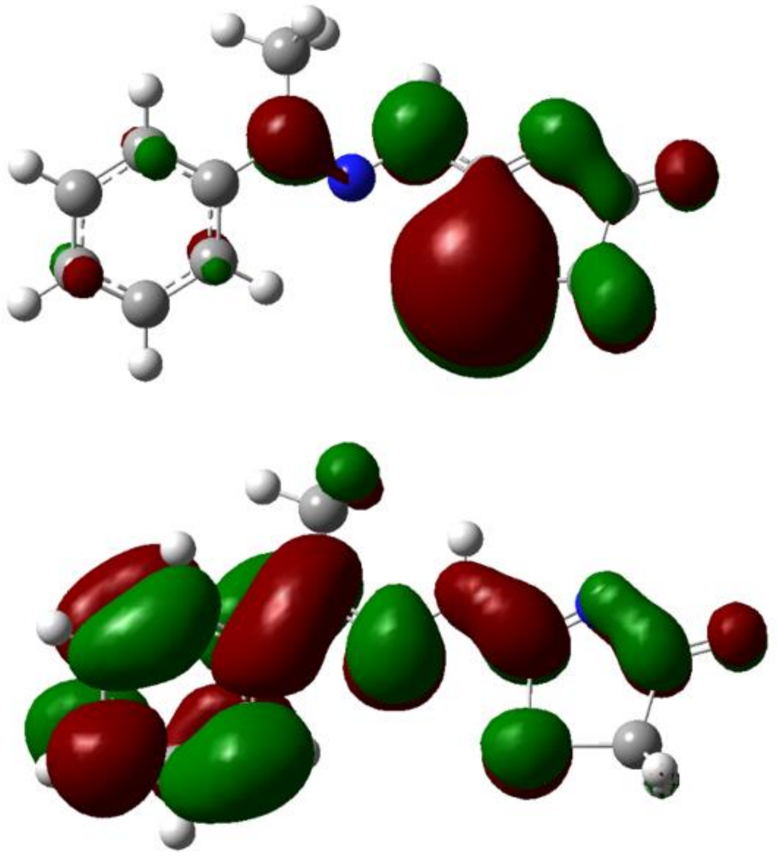

(a)

(b)

Figure S22. Orbitals in HOMO (top) and LUMO (bottom) in **DKI24** endo conformation.

Table S1. HOMO- LUMO gap, hardness, and softness of lowest in energy endo and exo conformers .

	DKI21 endo	DKI21 exo	DKI24 endo	DKI24 exo
H-L	4.302	4.237	4.549	4.364
η	2.151	2.118	2.275	2.182
μ	-4.331	-4.160	-4.349	-4.144
S	0.465	0.472	0.440	0.458
